# Optimization of Approaches to Analysis of Lignin by Thermal Decomposition

**DOI:** 10.3390/polym15132861

**Published:** 2023-06-28

**Authors:** Sergey Pokryshkin, Yuliya Sypalova, Artem Ivahnov, Aleksandr Kozhevnikov

**Affiliations:** Core Facility Center “Arktika”, Northern (Arctic) Federal University named after M.V. Lomonosov Northern Dvina Emb., 17, 163002 Arkhangelsk, Russia; serge.physchem@yandex.ru (S.P.); yuliya.popova01@mail.ru (Y.S.); ivahnov-tema@yandex.ru (A.I.)

**Keywords:** lignin, Py–GC/MS, thermal destruction, HSQC NMR

## Abstract

The ratio of monomeric units is one of the main characteristics of lignin, which affects the possibilities and strategies for further processing. Pyrolytic and thermal desorption decomposition of lignins followed by mass detection of macromolecule fragments are the most common methods for determining the amount of lignin structural units. Two methods of thermal decomposition of lignin were studied: thermal desorption–GC/MS (TD–GC/MS) and pyrolysis–GC/MS (Py–GC/MS). It was noted that, when using different thermal decomposition modes, the composition of the products changes, which affects the accuracy of determining the amount of lignin structural fragments. This article investigated the influence of the sample weight, the thermal decomposition temperature, and the duration of the process in various modes on the quantitation of the lignin structural units. The optimal process conditions were established. It was shown that the DS–Py–GC/MS with cryofocusing, a sample weight of 0.2–0.4 mg, and heating from 50 to 400 °C at a rate of 120 °C/min are preferable. The HSQC NMR was used as a comparison method to obtain the content of the S/G/H units. The results showed the applicability of the proposed approaches to hardwood lignins close to native.

## 1. Introduction

Nowadays, biorefining technologies are increasingly developing that involve processing lignin into useful products along with cellulose [1]. In the classical sense, lignin is a large-tonnage pulp and paper industry waste, which is mainly used as a fuel for energy recovery [2]. Approximately 95% of technical lignin is burned in thermal power plants, and only 5% is used for the following modification [3]. Lignin valorization is limited by the problems of strong structure modification during the technological processes, which does not allow the implementation of direct industrial use and requires complex biorefining systems. In this regard, lignins are often considered low-value products, and their refining is expensive. Therefore, lignin valorization should be based on a strong knowledge of its structure. In the last few years, new integrated approaches to the production of lignin-derived products have been actively developed with eco- and cost-efficient methods [4].

According to modern concepts, lignin is an aromatic heteropolymer with an irregular structure. This assumption is based on the lability of the lignin structure, which is formed during the enzymatic radical dehydrogenation polymerization of three monolignols (coniferyl, sinapyl, and p-coumaryl alcohols) with the formation of various types of bonds between the phenylpropane structural units. Thus, the lignin macromolecule consists of guaiacyl (G), syringyl (S), and p-hydroxyphenyl (H) structural units. The diversity of the bonds between the structural units as well as the possibility of partial oxidation with the formation of aldehyde, ketone, and carboxyl functional groups make it possible to acquire an extremely complex branched structure [5]. The ratio of the monomeric units is one of the main characteristics of lignin, which affects the possibilities and strategies for further processing. This ratio is expressed as S:G:H (or S/G/H). This characteristic is no less important for assessing native lignin since the content of the monomeric units depends on the type of plant biomass [6]. For example, softwood lignins consist mainly of G units (80–90%), hardwood lignins contain both G units (25–50%) and S units (50–70%), and non-wood lignins contain a mix of aromatic units: G (25–50%), S (25–50%), and H (10–25%) [7].

Various spectroscopic methods, such as FTIR, FT–Raman, UV, and NMR spectroscopy, are used to determine the lignin structural units. Given that most methods for the S/G/H determination have specific disadvantages, many researchers use a combination of these methods and compare the results of each [8,9]. Using FTIR or FT–Raman, the relative intensity of the corresponding spectral bands is measured with respect to the aromatic ring vibrations of the phenylpropane skeleton at 1606, 1510, and 1425 cm^−1^ [10]. However, the use of these methods is limited by the overlap of the vibration bands of different bond types, which makes it difficult to accurately determine the S, G, and H units. In addition, the overlap of the absorption bands of the lignin macromolecule bonds and residual carbohydrate bonds leads to an overestimation of S/G/H. The spectral sensitivity to water also has an interfering effect [8,11,12,13].

Nuclear magnetic resonance spectroscopy (NMR) is one of the leading spectroscopic methods in chemistry related to non-destructive methods of analysis. Currently, many researchers use the HSQC NMR technique to analyze the lignin macromolecule structure [14,15,16]. This method is one of the most accurate in terms of the quantitation of the lignin structural units. However, an important parameter is that the samples should be soluble in the NMR solvents, which is not always feasible. In addition, lignin quantitation is difficult due to spectra overlap, lack of spectral resolution especially in the lignin region, and lack of sensitivity [11].

The pyrolysis–gas chromatography/mass spectrometry is widely used today to quantify the S/G/H units [17,18,19]. However, care must be taken in interpreting the results due to the nature of the pyrolytic process, as the feedstock may not always degrade to the expected or desired products [20]. When using this method, researchers apply various modes of thermal decomposition. There is a strong influence of the pyrolysis temperature on the composition of the volatile products. The pyrolysis parameters affect the composition of the products detected during the analysis as well as the content of the lignin monomeric units, which can lead to incorrect conclusions about the macromolecule structure [19,21]. The researchers note that the S/G/H values obtained with Py–GC/MS differ from those of the same samples obtained with HSQC NMR [9,22]. Therefore, for the correct interpretation of the data obtained, an «arbitration» method is needed, which gives a rather correct result.

Nevertheless, in our opinion, pyrolytic decomposition methods should not be refused in the lignin structure issues, since the Py–GC/MS method is quite simple and express, and the optimization of its parameters will make it possible to eliminate inaccuracies in the conclusions about the lignin macromolecule structure.

The main purpose of this study was to identify factors affecting the performance of thermal desorption and pyrolytic decomposition of lignin macromolecules. The optimal parameters for determining the exact content of the monomeric units in lignin were established. The HSQC NMR was used as a comparison method to obtain the S/G/H. Selection of this method as “arbitration” is due to the fact that HSQC NMR gives an accurate and reproducible result and also works on completely different physical principles.

This work was aimed at optimizing the approach to determining the content of the S/G/H units primarily in lignins close to native ones. Birch wood is traditionally used in the pulp and paper industry, and hardwood lignins have great potential for subsequent valorization [9]. Three types of organosolv birch lignin were used as objects of study—milled wood lignin (MWL), alkaline ethanol lignin (EtOH), and dioxane lignin (DL).

Two methods of thermal decomposition of lignin were studied: thermal desorption–GC/MS (TD–GC/MS) and pyrolysis–GC/MS (Py–GC/MS). The decomposition parameters for these methods were selected separately. Most of the experiments were carried out with the MWL sample. The specified experimental parameters were tested on other organosolv lignin preparations.

## 2. Materials and Methods

### 2.1. Materials

Silver birch (*Bétula péndula*, 40 years old) wood harvested from the Arkhangelsk forestry was studied. The trunk part of the tree was debarked and then sawn using a grease-free saw into cross-sections with a thickness of 20 mm. The wood samples were cut to a fraction of approximately 10 × 10 mm. The wood chips were milled using an analytical knife mill IKA A11 Basic (Staufen, Germany). The sawdust was screened to collect 0.5–0.12 mm particle size and air dried at room temperature. Extractives were removed using acetone extraction of the sawdust in a Soxhlet apparatus. The amount of extractives in the original birch sawdust was 7.6%. The extractive-free birch sample contained 39.2% cellulose, 39.3% hemicelluloses, and 21.2% lignin (20.9% Klason lignin, 0.3% acid-soluble lignin), determined according to NREL’s standard analytical method [23].

All chemicals used were purchased from Aldosa (Moscow, Russia). All chemicals were of analytical grade and were used without further purification. Pure water was obtained with a Millipore Simplicity UV system (Merck KGaA, Molsheim, France).

### 2.2. Isolation of Lignins

#### 2.2.1. Milled Wood Lignin

Crude milled wood lignin was isolated based on the classical method [24] with some modifications. The dewaxed birch powder (15 g) was milled in a planetary ball mill (Retsch, Haan, Germany) equipped with a 250 mL tungsten carbide bowl containing 45 balls of 1 cm diameter in contrast to the vibratory ball mill utilized in Bjorkman’s protocol. The milling was conducted for a total milling time of 8 h (30 min running, 15 min pause in between) at 550 rpm. The wood meal obtained was extracted with dioxane (96% *v*/*v*) with a solid-to-liquid ratio of 1:10 (g/mL) at room temperature for 24 h under sufficient shaking. The solvent was then rotary evaporated at 40 °C. To remove the traces of dioxane, a few drops of water were added to the solid and evaporated again. The procedure of extraction was repeated three times. Finally, the solid matter was dried in a vacuum oven at 40 °C to obtain the MWL preparation. The yield of MWL was 49% per Klason lignin.

#### 2.2.2. Alkaline Ethanol Lignin

The EtOH lignin was obtained during the pulping with an aqueous alcohol solution of sodium hydroxide [25]. The dewaxed birch powder was treated with 40% ethanol solution containing 5% NaOH at a solid-to-liquid ratio of 1:6 (g/mL). The duration of heating from 25 °C to 100 °C was 20 min, and the duration of the isothermal stage at 100 °C was 1 h. Then, the temperature was increased to 150 °C, and the duration of the isothermal stage at 150 °C was 2 h. The duration of cooling at the end of the process was 20 min. The pulp mass and liquid lignin stream were separated on a Buchner funnel and washed with deionized water until the filtrate was neutral. The lignin solution was passed through a filtering crucible (pore size 100–160 µm) to separate the residual cellulose fibers. The filtrate was collected and adjusted to pH 3.0 with H_2_SO_4_. The precipitated lignin was repeatedly washed with pure water until the washings were neutral, centrifuged, and dried in a vacuum oven at 35 °C to the constant mass. The yield of EtOH lignin was 63% per Klason lignin.

#### 2.2.3. Dioxanelignin

The DL extraction procedure was carried out according to our previous protocol [26] based on the Pepper method. The dewaxed birch powder was suspended in the dioxane solution (9:1 *v*/*v*) containing 0.2 N HCl and refluxed for 2 h under a nitrogen flow. The solid-to-liquid ratio was 1:8. Then, the liquid lignin stream was separated on a Buchner funnel, neutralized with Na_2_CO_3_, and filtered again. The filtrate was concentrated on a rotary evaporator. The concentrated liquid was poured dropwise into water to precipitate the lignin and further centrifuged. The lignin precipitate was washed and dried in a vacuum oven at 40 °C. The crude DL was dissolved in dioxane and precipitated dropwise in diethyl ether with constant stirring to remove impurities. The precipitate was filtered through a nylon membrane, washed with ether until the washings were colorless, and dried in a vacuum oven at 40 °C. The yield of DL was 47% per Klason lignin.

### 2.3. Analytical Methods

#### 2.3.1. NMR Spectroscopy

NMR spectra were recorded on a Bruker Avance III 600 MHz spectrometer at 25 °C according to the literature methods [27,28]. Approximately 80 mg of lignin sample was dissolved in 0.6 mL of DMSO-d6. The 2D HSQC (heteronuclear single quantum coherence) experiments used the Bruker’s sensitivity-enhanced pulse program «hsqcetgpsisp2» with echo/antiecho detection and gradient adiabatic pulses that utilizes shaped pulses for all 180° pulses on the proton channel. This sequence is considered to be more sensitive and makes it possible to obtain simpler spectra in which all contours are positive. The DQD acquisition mode was used. Spectral widths of 6600 Hz (from 11 to 0 ppm) and 32,448 Hz (from 215 to 0 ppm) were for the ^1^H- and ^13^C-dimensions, respectively. The acquisition time of 77.6 ms was set for the ^1^H-dimension, and 36 scans per block were collected using the 1024 collected complex points. For the ^13^C-dimension, the acquisition time was 3.94 ms, and 256 time increments were recorded. An interscan delay of 2.0 s was set, and the total acquisition time was 5 h 50 min. The average value for one-bond J coupling between protons and carbons (^1^J_CH_) used was 145 Hz. The 2D HSQC NMR data were manipulated with 1024 × 1024 data points applying the Qsine function for both the ^1^H and ^13^C dimensions. The DMSO peak at δC/δH 39.5/2.49 ppm was used for the spectra calibration.

The cross-peaks were assigned based on the literature data [27,28,29,30]. A semi-quantitative analysis of the volume integrals of the HSQC correlation peaks was performed [27,31]. The 2D HSQC NMR method was mainly used to obtain an «arbitration» value of the S/G/H. The aromatic/unsaturated (δC/δH 90–130/6.0–7.4 ppm) regions of the HSQC NMR spectra of MWL, DL, and EtOH are shown in Figure S1. The accuracy of 2D HSQC quantitative results depends on the resolution of the signals representing the different substructures. In the aromatic region, C_2,6_–H_2,6_ correlations from S units, C_2_–H_2_ correlations from G units, and C_2,6_-H_2,6_ correlations from H units were used to estimate the content of the monomeric units. The quantity of the different lignin signals was normalized using an assumption of:

G + S + H = G_2_ + S_2,6_/2 + H_2,6_/2 = 100 Ar (1)

#### 2.3.2. Thermal Desorption

The Shimadzu TD-20 thermal desorption system was used for the low-temperature, slow-heat-rate thermal decomposition of lignin. The GC/MS analysis of the lignin decomposition products was performed using a Shimadzu QP-2010 Plus gas chromatograph–mass spectrometer (Kyoto, Japan). The thermal desorption parameters were as follows: helium was used as the carrier gas flowing at 30 mLmin^−1^, and the volatile and semi-volatile products of lignin decomposition were trapped on the Tenax TA sorbent (Sigma Aldrich, Steinheim, Germany) in a cooling trap at −10 °C. The lignin sample weight was varied from 0.6 mg to 8.1 mg. Thermal decomposition was carried out in the range of 150–400 °C in 50 °C increments. The duration of the process was varied from 5 to 60 min.

The GC separation parameters were constant during all experiments. The GC was equipped with a HP-5MS fused-silica capillary column (30 m × 0.25 mm i.d., 0.25 µm f.t.). The gas flow was 1.0 mLmin^−1^ with the split ratio of 30:1. The GC oven temperature program began at 40 °C and then increased at a heating rate of 3 °C/min to 200 °C followed by a post-run program at 320 °C for 3 min. The MS was operated in EI mode (70 eV) at 230 °C. The components were identified using the NIST and Wiley libraries.

#### 2.3.3. Analytical Pyrolysis

Pyrolysis of the isolated lignins was performed with an EGA/PY-3030D pyrolyzer (Frontier Laboratories Ltd., Koriyama, Japan) connected to the same Shimadzu GC/MS QP-2010 Plus system that was described previously. The lignin sample weights were varied from 0.2 mg to 0.9 mg. The pyrolysis temperature was wide-ranged from 250 °C to 700 °C in 50 °C increments. The chemical composition of the lignin was analyzed in two modes: slow programmed heating (Double Shot) and fast uncontrolled heating (Single Shot).

In the double-shot (DS) mode, the sample was placed into the pyrolyzer furnace at a low temperature (50 °C), and then the temperature was raised at a given rate to the final one. After reaching the final temperature, the sample was removed from the furnace, the cryo-trap was turned off, and then the GC/MS analysis was performed. For the DS mode, the heating rate, sample weight, and the final pyrolysis temperature were varied. The carrier gas flow rate (50 mLmin^−1^), initial furnace temperature (50 °C), cryo-trap temperature (−198 °C), and GC separation parameters remained constant.

In the single-shot (SS) mode, the sample was placed into a preheated furnace at a fixed temperature and remained inside until the end of the pyrolysis time. The furnace temperature was varied. The sample weight was similar to the DS mode, and the pyrolysis time was 3 min (same as the optimal time for the DS mode). The cryo-trap was kept in a cold state for the duration of the pyrolysis; then, it was turned off, and GC/MS analysis was performed. The carrier gas flow rate, initial furnace temperature, cryo-trap temperature, and GC separation parameters were set as in the DS mode.

The GC separation parameters were the same as in the TD experiments and described above. The compounds were identified by comparing their mass spectra with those of the Wiley and NIST libraries as well as with the literature data [32,33].

The content of H, G, and S structures was calculated as a percentage of the sum of the areas of the phenylpropane thermal destruction products with one hydroxyl group. The content of polyphenolic structures (Poly-OH) was calculated as a percentage of the sum of the areas of all thermal destruction products with one or more hydroxyl groups coupled with one aromatic ring. Thus, the percentage of Poly-OH structures cannot be added to the percentage of HGS structures. It was used to assess the intensity of the secondary thermal degradation reactions.

## 3. Results and Discussion

The products of the thermal decomposition of lignin are listed in Table S1. The volatile lignin decomposition products were divided into four groups: H—phenolic compounds without methoxyl functional groups; G—C3-methoxy-substituted phenolic compounds; S—C3,5-methoxy-substituted phenolic compounds; and Poly-OH—polyphenolic compounds with two or more phenolic hydroxyl groups.

The first three groups of compounds are the primary products of lignin pyrolysis, and their formation begins at relatively low temperatures (from 200 °C) as a result of the thermal cleavage of the bonds of the lignin macromolecule. Polyphenolic compounds are products of secondary reactions formed at higher temperatures (400 °C and above).

**Figure 1:**
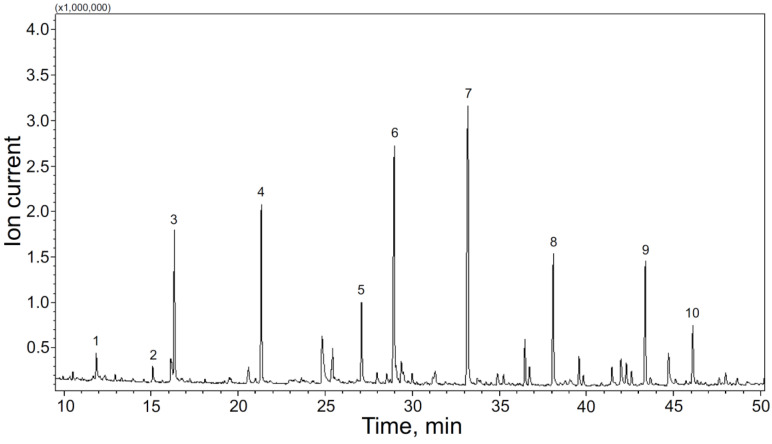
Chromatogram of the MWL volatile pyrolysis products: 1—Phenol; 2—2-methylphenol; 3—2-methoxyphenol; 4—2-methoxy-4-methylphenol; 5—2-Methoxy-4-vinylphenol; 6—2,6-dimethoxyphenol; 7—2,6-dimethoxy-4-methylphenol; 8—2,6-dimethoxy-4-vinylphenol; 9—2,6-dimethoxy-4-(2-propenyl)phenol; 10—2,6-dimethoxy-4-(2-oxopropyl)phenol.

Among the H, G, and S groups, the main compounds that predominate in the pyrolysis products can be distinguished (Figure 1). These are phenol (peak 1 at the Figure 1) and 4-methylphenol (peak 2) from the H group; 2 methoxyphenol (guaiacol, peak 3) and 4-methyl-2-methoxyphenol (creosol, peak 4) from the G group; and syringol (peak 6) and 4-methyl-2,6-dimethoxyphenol (4-methylsyringol, peak 7) from the S group.

Thus, the main products of each group were its simplest compound (phenol with a characteristic functional substituent) and the 4-methyl derivative of this compound. This can be explained by the abundance of β-O-4 bonds in lignin, which are cleaved first during thermal decomposition.

### 3.1. Optimization of TD–GC/MS Analysis Parameters for Lignin Decomposition

#### 3.1.1. Effect of Temperature on the Thermal Decomposition

**Figure 2:**
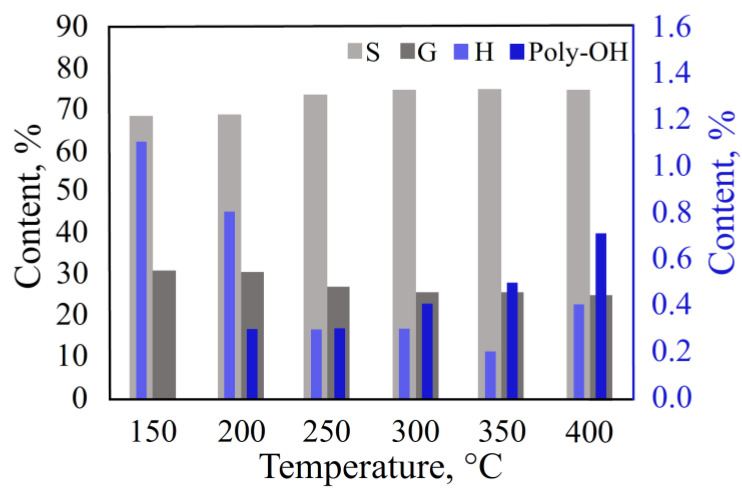
Effect of temperature on the thermal decomposition of birch MWL.

According to the data obtained (Figure 2, Table S2), the thermal degradation of lignin occurred at temperatures above 200 °C. In the temperature range of 250–400 °C, the dispersion of values for the G and S units was less than 5%.

However, at temperatures above 300 °C, a significant yield of by-products, such as catechol, methylcatechol, and pyrogallol, resulting from the secondary reactions between the thermal decomposition products, was noted (Figure S2). On the other hand, decreasing the temperature from 300 °C to 250 °C significantly reduces the yield of thermal degradation products. Thus, the optimal process temperature cannot be determined without an arbitration method.

According to the HSQC NMR data, the content of the lignin monomeric units was as follows: S—69.8%, G—29.5%, and H—1.3%. A comparison of the data obtained with the two different methods showed that the closest content of lignin decomposition products was observed at 200 °C. Therefore, based on the experimental data, the process temperature should be set from 200 °C to 250 °C.

#### 3.1.2. Effect of Sample Weight

**Figure 3:**
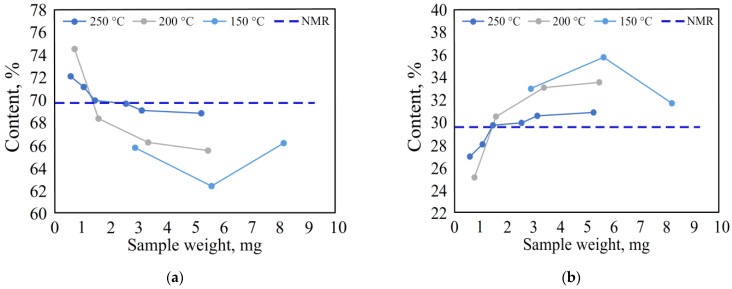
Effect of sample weight on the thermal decomposition of birch MWL: (**a**) on the content of the S units; (**b**) on the content of the G units.

The lignin sample weight was varied from 0.6 to 5.2 mg for decomposition at 200 °C and 250 °C and from 2.8 to 8.1 mg for decomposition at 150 °C due to much lower decomposition yield at low temperature. For the MWL samples at a weight of 0.6–5.2 mg, the content of the G and S units differed from the NMR method by no more than 1 %. Sample weights outside this range led to an underestimation of the G units and an overestimation of the S units. The results showed (Figure 3) that a sample weighing 1.5–3 mg should be taken.

#### 3.1.3. Effect of TD Duration

**Figure 4:**
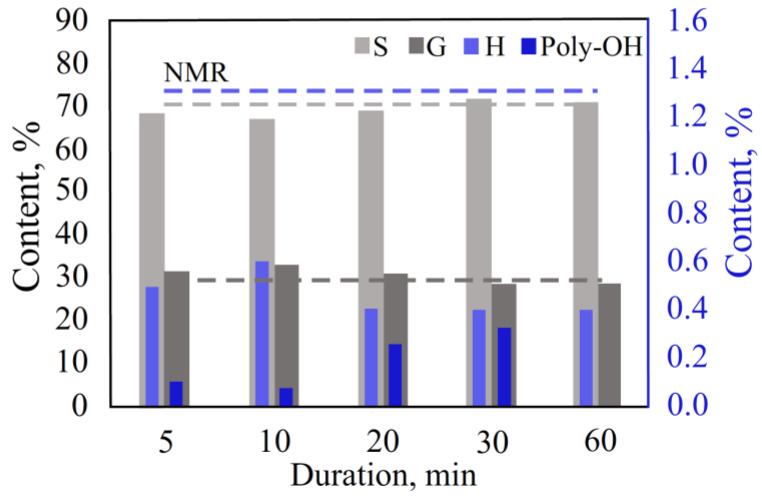
Effect of thermal desorption duration.

The influence of the thermal decomposition duration in the TD mode per each lignin unit (S/G/H) was studied (Figure 4, Table S3). At a decomposition time of less than 20 min, the results were unstable, most likely due to uneven heating of the tube with the sample. With a TD duration of 20–60 min, the results of the analysis changed insignificantly.

The content of the S and G units obtained at 20 and 30 min decomposition times was the closest to the NMR data. However, it was not possible to achieve the exact content of the H units due to their relatively low content in the sample.

Based on the experimental data, specific TD conditions were established. The recommended temperature for the process is 200–250 °C, and the duration is 20–30 min with a sample weight of 1.5–3 mg.

### 3.2. Optimization of Py–GC/MS Analysis Parameters for Lignin Decomposition

#### 3.2.1. Heating Rate in the Double-Shot Mode

**Figure 5:**
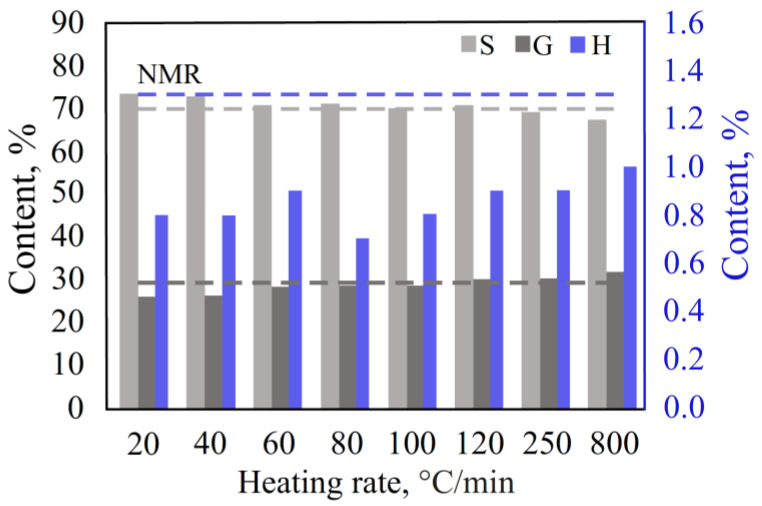
Effect of heating rate in the DS mode.

The DS mode is a smooth programmable heating of the sample in an inert gas atmosphere. The heating rate was varied in the range of 20 °C/min (the pyrolysis time was 17.5 min) to 800 °C/min (the pyrolysis time was 0.5 min). The initial temperature was 50 °C, and the final temperature was 400 °C for all runs. The results showed (Figure 5, Table S4) that the decrease in the heating rate reduced the content of the G and H units and increased the content of the S units in the pyrolysis products. The contents of the S/G/H units closest to the HSQC NMR data were obtained at a heating rate from 100 to 120 °C/min (pyrolysis time was 3 min).

#### 3.2.2. Effect of Sample Weight in the DS Mode

In the smooth programmable heating mode with the optimal pyrolysis time established on the previous step, the MWL sample weights were varied from 0.2 to 0.9 mg.

**Figure 6:**
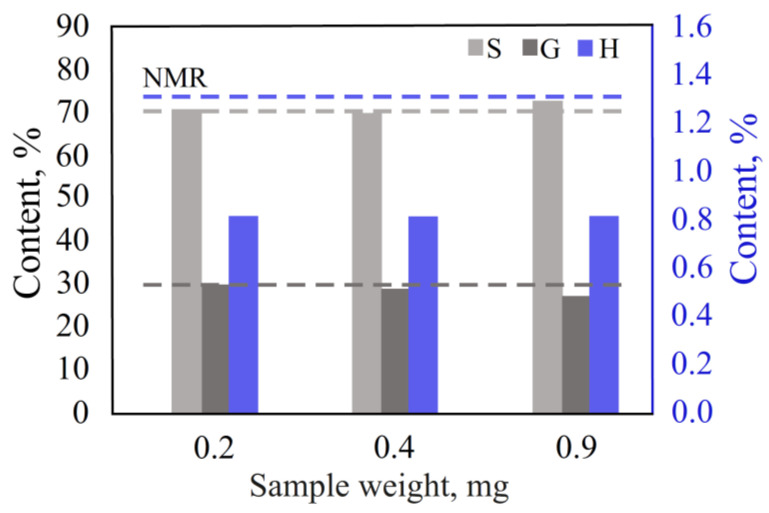
Effect of sample weight in the DS mode.

In that sample weight range, the results showed (Figure 6, Tables S1 and S5) the detection of all chromatographic peaks for all required fragments of the lignin macromolecule.

However, the detector was «overloaded» and/or the peak shapes were deformed with the increasing of the sample weight, which declined the chromatographic separation and made peak integration difficult. Table S6 lists the peak shape parameters for the volatile pyrolysis products with the maximum peak area—syringol and 4-methylsyringol. With the sample weight increasing from 0.2 to 0.9 mg, the ratio of the peak area to the peak height (A/H) increased from 6.8–8.5 to 12.3–13.3, full width at half maximum (FWHM) increased from 0.10 to 0.20–0.21, asymmetry factor (AsF) for 4-methylsyringol decreased from 0.61 to 0.22 and from 1.71 to 0.26 for syringol, and tailing factor (TF) changed from 0.86 to 0.62 for 4-methylsyringol and from 1.63 to 0.64 for syringol. All of these changes showed the distortion of the peak shape with the sample weight increasing.

On the other side, the content of the S/G/H units obtained with a sample weight of 0.2–0.4 mg were the closest to the NMR data. Thus, a lignin sample weight below 0.4 mg was set as optimal.

#### 3.2.3. Effect of Temperature in the DS Mode

**Figure 7:**
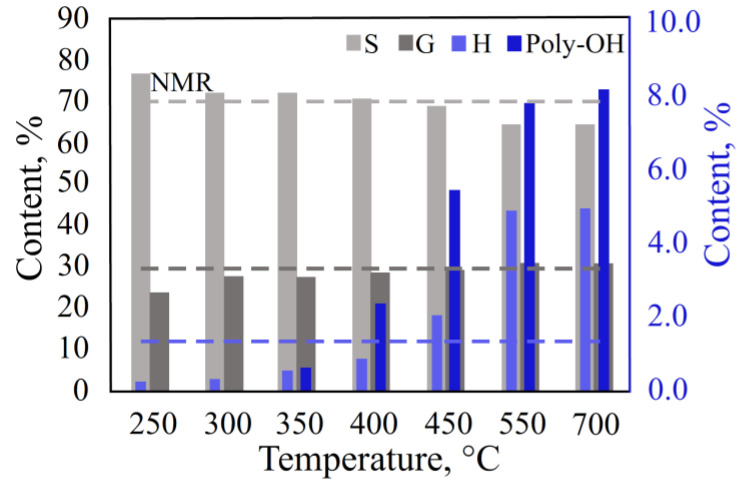
Effect of temperature in the DS mode.

In the smooth programmable heating mode, final pyrolysis temperatures from 250 °C to 700 °C were used (Figure 7, Table S7). The peak area at 250 °C was reduced, and the relative content of S/G/H was not correlated with the NMR data. This suggests that the lignin decomposition was insufficient, and the temperature of 250 °C was not enough for the pyrolysis processes. At temperatures of 400 °C and 450 °C, the content of the S, G, and H units in the pyrolysis products were the closest to the NMR data. However, at temperatures above 400 °C, the yield of by-products, such as catechol, methylcatechol, and pyrogallol, resulting from the secondary reactions between the thermal decomposition products, was increased. Therefore, the final pyrolysis temperature should be set equal to 400 °C.

The recommended conditions for the pyrolysis process in the DS mode are as follows: smooth heating from 50 °C to 425 °C with a pyrolysis time of 3 min (heating rate 120 °C/min) and a sample weight of 0.2–0.4 mg.

#### 3.2.4. Effect of Temperature in the Single-Shot Mode

The SS mode is a fast, uncontrolled heating of the sample in an inert gas atmosphere. For the experiment, several pyrolytic decomposition parameters were set the same as in the DS mode (3 min pyrolysis time and 0.2–0.4 mg of sample). The pyrolysis process in the temperature range (final temperature) from 250 °C to 700 °C was studied.

**Figure 8:**
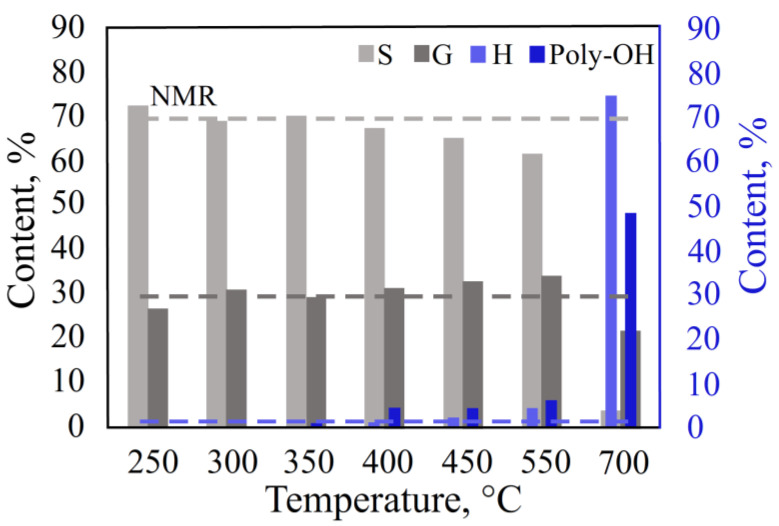
Effect of temperature in the SS mode.

The results showed (Figure 8, Table S8) that, at 250 °C, the yield of decomposition products decreased, and the content of the S/G/H units poorly correlated with the NMR data. This suggests that the lignin decomposition was insufficient and affected only the most labile bonds. At temperatures of 400 °C and above, the S/G/H contents deviated from the NMR data, and the yield of thermal degradation by-products (catechol, methylcatechol, and pyrogallol) increased. At a temperature of 350 °C, the results were closest to the arbitration NMR data. At a temperature of 400 °C, the minimum deviation in the content of the H units was observed, and at 350 °C, the minimum deviation in the content of the G and S units was observed.

Based on the experimental data, specific parameters for lignin thermal decomposition were established (Table 1). They are suitable for the reliable determination of lignin monomeric units and are focused on the study of lignins close to native ones.

### 3.3. Application of the Specified Thermal Decomposition Conditions for Various Lignin Preparations

It has been proposed that, under the established conditions, the decomposition of various lignin preparations into monomers would occur in different ways. Therefore, data on the content of the monomeric units for three types of organosolv lignins (MWL, EtOH, and DL) were obtained (Table 2).

It can be concluded that the established modes of thermal decomposition are applicable to all studied lignin samples. Based on Table 2, the difference between the data obtained with thermal decomposition and NMR was calculated (Table 3).

Minimal differences from the NMR data were noted for the DS mode. This could be related to the secondary reactions weakening due to slow heating as well as the continuous removal of volatile degradation products by the carrier gas flow, which reduces their concentration in the vapor phase. The data obtained with the SS–Py–GC/MS and TD–GC/MS methods are inferior to the NMR data. The differences between the SS and DS modes are related to the various secondary reaction rates under the different heating rates of the sample. In the SS mode with fast uncontrolled heating, a denser cloud of volatiles is formed in which secondary reactions are enhanced.

The differences between the data obtained with TD and NMR could be due to the complex design of the TD (long pipes, six port valve, cryo-trap with sorbent) and using Tenax sorbent in a cryo-trap. Despite the deactivated surfaces of the pipes and valves, they probably affected some reactive products. This can lead to sorption/condensation of the thermal degradation products and enhance secondary reactions between them.

Therefore, to quantify the S/G/H with the highest agreement with the NMR data, it is preferable to use the DS–Py–GC/MS with cryofocusing, a sample weight of 0.2–0.4 mg, and heating from 50 to 400 °C at a rate of 120 °C/min.

## 4. Conclusions

The content of the lignin monomeric units (S/G/H) is an important characteristic of the lignin macromolecule, and its measured value will be affected by a change in the composition of the final pyrolysis products. In the quantitation of the S/G/H units of lignins with thermal decomposition methods, the composition of the final products is influenced by such parameters as the sample weight, the temperature of the process, the heating rate, and the duration of the process.

To find the optimal parameters for the thermal decomposition of lignins, the HSQC NMR spectroscopy was used as an arbitration method. The decomposition parameters for thermal desorption–GC/MS (TD–GC/MS) and pyrolysis–GC/MS (Py–GC/MS) methods were clarified. The parameters of the pyrolytic decomposition of lignin were set in two modes: slow programmed heating (Double Shot) and fast uncontrolled heating (Single Shot).

The optimal parameters of the thermal desorption at which the highest agreement with HSQC NMR was observed were as follows: decomposition temperature 200–250 °C with a process duration of 20–30 min and a sample weight of 1.5–3 mg.

The recommended conditions for the pyrolysis process in the DS mode were as follows: smooth heating from 50 °C to 425 °C with a pyrolysis time of 3 min (heating rate 120 °C/min) and a sample weight of 0.2–0.4 mg.

For the pyrolysis in the SS mode, the recommended pyrolysis temperature is 350 °C, and the pyrolysis time and sample weight are as in the DS mode—3 min and 0.2–0.4 mg, respectively.

The established parameters of thermal decomposition of lignin are applicable to all low-changing hardwood lignins isolated under relatively mild delignification conditions. The proposed method can be used to further explore herbal lignin in order to enrich the quantitative law of monomer structure.

## Figures and Tables

**Table 1:** Optimal parameters of lignin thermal decomposition.

Parameter	Py–GC/MS		TD–GC/MS
	Single Shot	Double Shot	
Final temperature, °C	350	400	200–250
Heating rate, °C/min	–	120	–
Process duration, min	1	3	30
Sample weight, mg	0.2–0.4	0.2–0.4	1.5–3.0

**Table 2:** Relative content of lignin structures at different thermal decomposition modes, %.

Unit ^1,2^	Py–GC/MS		TD–GC/MS	HSQC NMR
	Single Shot	Double Shot		
S_MWL_	70.2	70.5	67.6	69.8
S_EtOH_	67.8	70.4	75.9	72.6
S_DL_	69.1	70.7	66.5	71.4
G_MWL_	29.4	28.7	31.8	29.5
G_EtOH_	31.5	28.7	23.2	26.6
G_DL_	30.4	29.1	33.5	27.9
H_MWL_	0.4	0.8	0.6	1.3
H_EtOH_	0.8	0.9	0.9	1.6
H_DL_	0.3	0.3	0.0	1.5
Poly-OH_MWL_	4.6	2.4	0.2	-
Poly-OH_EtOH_	1.2	3.4	0.0	-
Poly-OH_DL_	2.3	1.9	0.0	-

^1^ S + G + H = 100%; ^2^ Poly-OH—% of the sum of the areas of all thermal destruction products with one or more hydroxyl groups coupled with one aromatic ring. It is a measure of secondary reactions of thermal destruction.

**Table 3:** Average error of thermal destruction methods relative to the HSQC NMR method.

Unit	Py–GC/MS		TD–GC/MS
	Single Shot	Double Shot	
S	2.50	1.20	3.47
G	2.50	1.37	3.77
H	0.97	0.80	0.97

## Data Availability

The data reported in this study are publicly available as Open Access article and Supplementary Materials.

## Supplementary Materials

The following supporting information can be downloaded at: https://www.mdpi.com/article/10.3390/polym15132861/s1, Figure S1: The aromatic/unsaturated regions of the HSQC NMR spectra of (a) MWL, (b) DL, and (c) EtOH; Figure S2: Pyrograms of the by-products resulting from secondary reactions at 250, 350, and 700 °C: (a) catechol, (b) methylcatechol, and (c) pyrogallol; Table S1: Products of thermal decomposition of lignin; Table S2: Effect of temperature on the thermal decomposition; Table S3: Effect of thermal desorption duration; Table S4: Effect of heating rate in the DS mode; Table S5: Effect of sample weight in the DS mode; Table S6: Peak shape parameters; Table S7: Effect of temperature in the DS mode; Table S8: Effect of temperature in the SS mode.

## References

[1] Hu J., Zhang Q., Lee D.J. (2018). Kraft lignin biorefinery: A perspective. Bioresour. Technol..

[2] Yuan T.Q., Xu F., Sun R.C. (2013). Role of lignin in a biorefinery: Separation characterization and valorization. J. Chem. Technol. Biotechnol..

[3] Sun R.C. (2020). Lignin source and structural characterization. ChemSusChem.

[4] Balakshin M.Y., Capanema E.A., Sulaeva I., Schlee P., Huang Z., Feng M., Borghei M., Rojas O.J., Potthast A., Rosenau T. (2021). New opportunities in the valorization of technical lignins. ChemSusChem.

[5] Boerjan W., Ralph J., Baucher M. (2003). Lignin biosynthesis. Annu. Rev. Plant Biol..

[6] Anderson E.M., Stone M.L., Katahira R., Reed M., Muchero W., Ramirez K.J., Beckham G.T., Román-Leshkov Y. (2019). Differences in S/G ratio in natural poplar variants do not predict catalytic depolymerization monomer yields. Nat. Commun..

[7] Li C., Zhao X., Wang A., Huber G.W., Zhang T. (2015). Catalytic transformation of lignin for the production of chemicals and fuels. Chem. Rev..

[8] Del Rio J.C., Gutiérrez A., Rodríguez I.M., Ibarra D., Martinez A.T. (2007). Composition of non-woody plant lignins and cinnamic acids by Py-GC/MS, Py/TMAH and FT-IR. J. Anal. Appl. Pyrolysis.

[9] Paulsen Thoresen P., Lange H., Crestini C., Rova U., Matsakas L., Christakopoulos P. (2021). Characterization of organosolv birch lignins: Toward application-specific lignin production. ACS Omega.

[10] Martín-Sampedro R., Santos J.I., Fillat Ú., Wicklein B., Eugenio M.E., Ibarra D. (2019). Characterization of lignins from *Populus alba* L. generated as by-products in different transformation processes: Kraft pulping, organosolv and acid hydrolysis. Int. J. Biol. Macromol..

[11] Lupoi J.S., Singh S., Parthasarathi R., Simmons B.A., Henry R.J. (2015). Recent innovations in analytical methods for the qualitative and quantitative assessment of lignin. Renew. Sust. Energ. Rev..

[12] Shao Z., Fu Y., Wang P., Zhang Y., Qin M., Li X., Zhang F. (2018). Modification of the aspen lignin structure during integrated fractionation process of autohydrolysis and formic acid delignification. Int. J. Biol. Macromol..

[13] Ona T., Sonoda T., Ito K., Shibatal M., Katayama T., Kato T., Ootake Y. (1998). Non-destructive determination of lignin syringyl/guaiacyl monomeric composition in native wood by Fourier transform Raman spectroscopy. J. Wood Chem. Technol..

[14] Balakshin M., Capanema E.A., Zhu X., Sulaeva I., Potthast A., Rosenau T., Rojas O.J. (2020). Spruce milled wood lignin: Linear, branched or cross-linked?. Green Chem..

[15] Zeng J., Helms G.L., Gao X., Chen S. (2013). Quantification of wheat straw lignin structure by comprehensive NMR analysis. J. Agric. Food Chem..

[16] Xu G., Shi Z., Zhao Y., Deng J., Dong M., Liu C., Murugadoss V., Mai X., Guo Z. (2019). Structural characterization of lignin and its carbohydrate complexes isolated from bamboo (*Dendrocalamus sinicus*). Int. J. Biol. Macromol..

[17] Kumar A., Biswas B., Saini K., Kumar A., Kumar J., Krishna B.B., Bhaskar T. (2021). Py-GC/MS study of prot lignin with cobalt impregnated titania, ceria and zirconia catalysts. Renew. Energ..

[18] Branco D.G., Santiago C., Lourenco A., Cabrita L., Evtuguin D.V. (2021). Structural Features of Cork Dioxane Lignin from *Quercus suber* L. J. Agric. Food Chem..

[19] Rencoret J., Gutiérrez A., Marques G., José C., Tobimatsu Y., Lam P.Y., Pérez-Boada M., Ruiz-Dueñas F.J., Barrasa M., Martínez A.T. (2021). New insights on structures forming the lignin-like fractions of ancestral plants. Front. Plant Sci..

[20] Reeves III J.B., Galletti G.C. (1993). Use of pyrolysis—Gas chromatography/mass spectrometry in the study of lignin assays. J. Anal. Appl. Pyrolysis.

[21] Kawamoto H. (2017). Lignin pyrolysis reactions. J. Wood Sci..

[22] Izaguirre N., Robles E., Llano-Ponte R., Labidi J., Erdocia X. (2022). Fine-tune of lignin properties by its fractionation with a sequential organic solvent extraction. Ind. Crops Prod..

[23] Sluiter A., Hames B., Ruiz R., Scarlata C., Sluiter J., Templeton D., Crocker D. (2008). Determination of Structural Carbohydrates and Lignin in Biomass. Lab. Anal. Proced..

[24] Bjorkman A. (1956). Studies on finely divided wood. Part I. Extraction of lignin with neutral solvents. Sven. Papperstidn..

[25] Navaee-Ardeh S., Mohammadi-Rovshandeh J., Pourjoozi M. (2004). Influence of rice straw cooking conditions in the soda–ethanol–water pulping on the mechanical properties of produced paper sheets. Bioresour. Technol..

[26] Popova Y.A., Shestakov S.L., Belesov A.V., Pikovskoi I.I., Kozhevnikov A.Y. (2020). Comprehensive analysis of the chemical structure of lignin from raspberry stalks (*Rubus idaeus* L.). Int. J. Biol. Macromol..

[27] Tarasov D., Schlee P., Pranovich A., Moreno A., Wang L., Rigo D., Sipponen M.H., Xu C., Balakshin M. (2022). AqSO biorefinery: A green and parameter-controlled process for the production of lignin–carbohydrate hybrid materials. Green Chem..

[28] Del Río J.C., Rencoret J., Prinsen P., Martínez Á.T., Ralph J., Gutiérrez A. (2012). Structural characterization of wheat straw lignin as revealed by analytical pyrolysis, 2D-NMR, and reductive cleavage methods. J. Agric. Food Chem..

[29] Balakshin M., Capanema E., Gracz H., Chang H.M., Jameel H. (2011). Quantification of lignin–carbohydrate linkages with high-resolution NMR spectroscopy. Planta.

[30] Ralph J., Landucci L. (2010). NMR of Lignins. Lignin and Lignans.

[31] Capanema E., Balakshin M., Katahira R., Chang H.M., Jameel H. (2015). How well do MWL and CEL preparations represent the whole hardwood lignin?. J. Wood Chem. Technol..

[32] Ralph J., Hatfield D. (1991). Pyrolysis-GC–MS characterization of forage materials. J. Agric. Food Chem..

[33] Meier D., Faix O., Lin S.Y., Dence C.W. (1992). Pyrolysis-gas chromatography-mass spectrometry of lignin. Methods in Lignin Chemistry.

