# Supplementary material for: Optimization of Approaches to Analysis of Lignin by Thermal Decomposition
Source: Polymers (Basel). 2023 Jun 28;15(13):2861. doi: 10.3390/polym15132861 (PMC10347083; doi:10.3390/polym15132861)
Supplement: Supplementary file 1 [file polymers-15-02861-s001.zip › polymers-2439256-supplementary.pdf]

Supplementary information

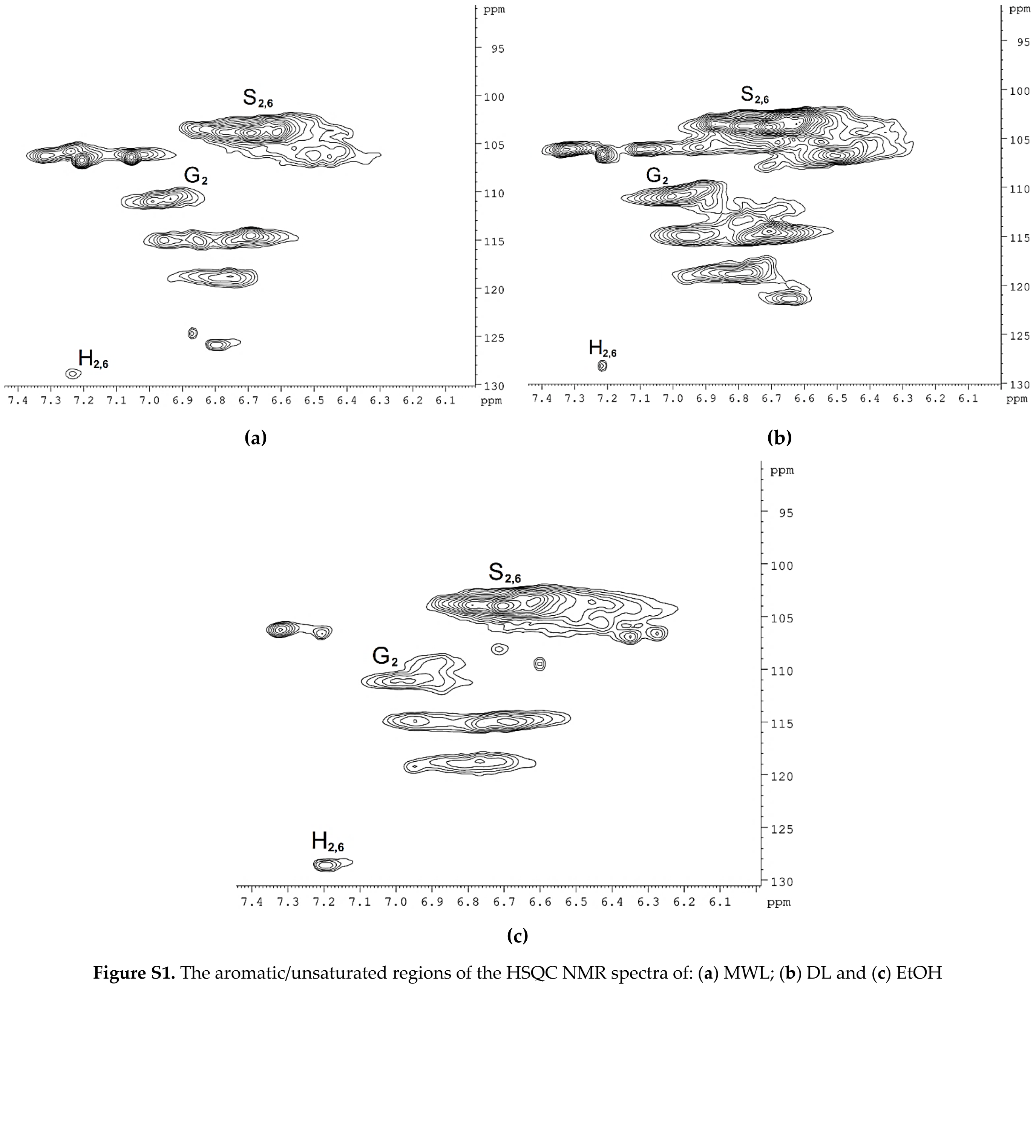

Figure S1. The aromatic/unsaturated regions of the HSQC NMR spectra of: (**a**) MWL; (**b**) DL and (**c**) EtOH

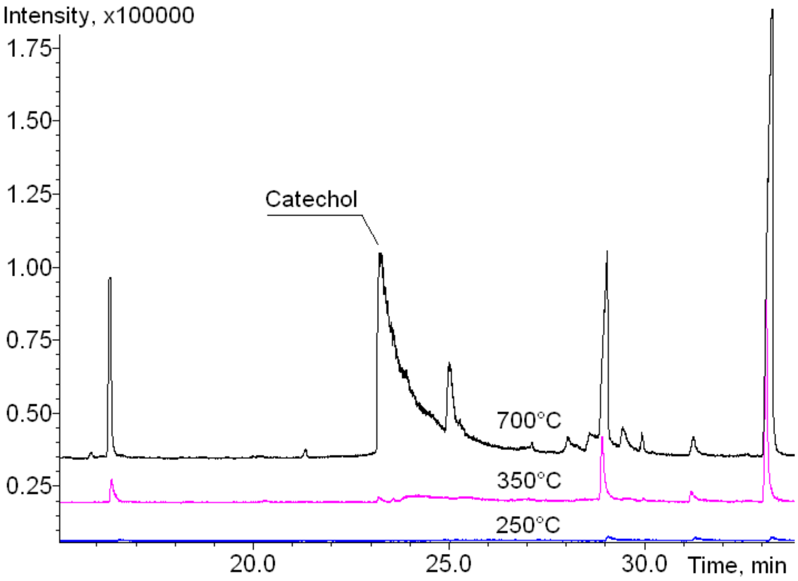

(a)

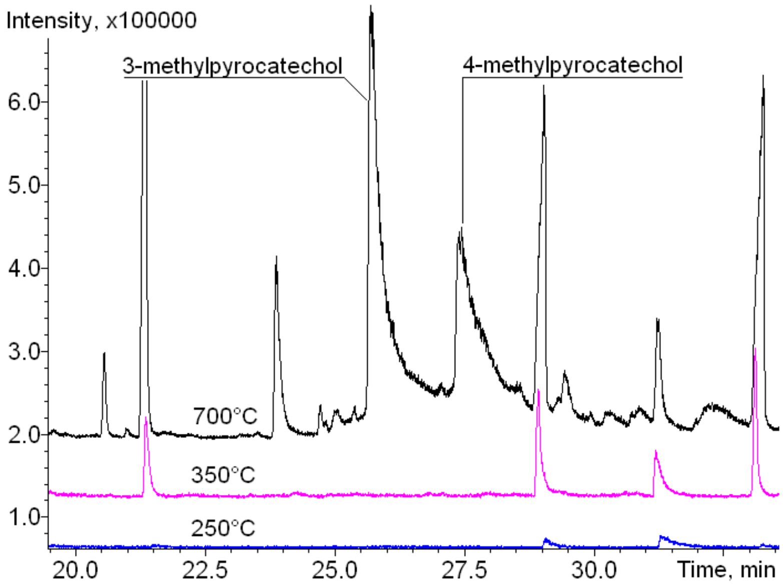

(b)

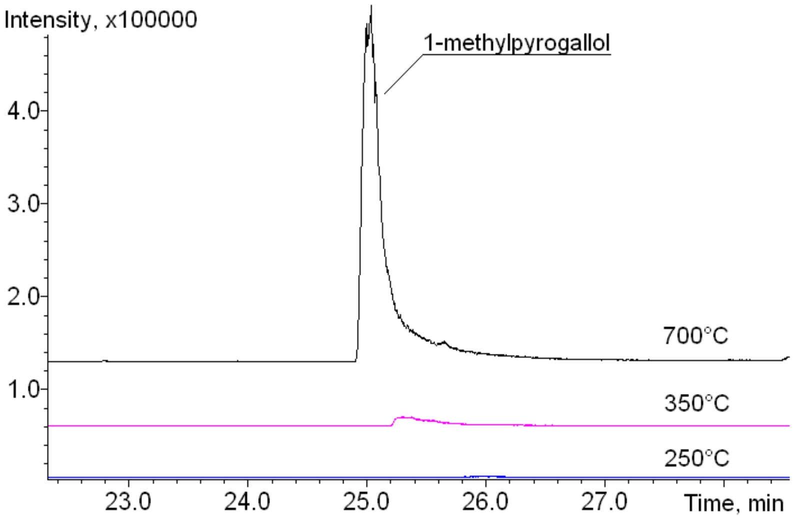

(c)

Figure S2. Pyrograms of the by-products, resulting from secondary reactions, at 250, 350 and 700 °C: (**a**) catechol; (**b**) methylcatechol; (**c**) pyrogallol

Table S1. Products of thermal decomposition of lignin

Name	Rt, min	Ri	Target Ion	Ref. ion	Group
***Phenol**	**11,942**	**971**	**94**	**66**	**H**
Phenol, 2-methyl-	14,981	1045	108	107	H
***Phenol, 4-methyl-**	**15,894**	**1067**	**107**	**108**	**H**
***Phenol, 2-methoxy- (Guaiacol)**	**15,952**	**1080**	**109**	**124**	**G**
Phenol, 2,6-dimethyl-	16,701	1098	107	122	H
Phenol, 2-methoxy-3-methyl	18,003	1130	123	138	G
Phenol, 2,5-dimethyl-	18,559	1135	107	122	H
Phenol, 2,4-dimethyl- + Phenol, 2,3-dimethyl-	18,895	1146	107	122	H
Phenol, 2-methoxy-5-methyl	19,773	1179	123	138	G
Phenol, 3-ethyl-	19,835	1183	107	122	H
Phenol, 4-methoxy-3-methyl	20,173	1187	123	138	G
Phenol, 4-ethyl-	20,213	1192	107	122	H
***Phenol, 2-methoxy-4-methyl (Creosol)**	**20,587**	**1196**	**123**	**138**	**G**
Catechol	21,965	1206	110	64	PolyOH
Phenol, 3-methoxy-	22,445	1235	124	94	G
Phenol, 4-ethyl-3-methyl-	22,748	1247	121	136	H
Phenol, 3,6-dimethoxy-	22,827	1260	154	139	S
Pyrogallol 1-methyl ether	23,862	1271	140	97	PolyOH
Phenol, 2,4,6-trimethyl-	23,903	1285	121	136	H
Phenol, 4-ethyl-2-methoxy- (Ethylguaiacol)	24,215	1294	137	152	G
Pyrocatechol, 3-methyl-	24,324	1299	124	123	PolyOH
Phenol, 4-ethyl-2-methoxy-	24,979	1317	137	152	G
2-Methoxy-4-vinylphenol	25,884	1322	150	135	G
Resorcinol	25,888	1322	110	82	PolyOH
Pyrocatechol, 4-methyl-	25,947	1325	124	123	PolyOH
Phenol, 3-methoxy-5-methyl	27,015	1341	138	107	G
Phenol, 3,4-dimethoxy-	27,196	1345	139	154	S
4-Propenylphenol	27,782	1363	134	133	H
Phenol, 3,4-dimethoxy-	27,826	1365	139	154	S
Acetophenone, 2-hydroxy-5-methoxy-	28,298	1383	151	166	G
Phenol, 2-methoxy-4-(2-propenyl)-	28,434	1385	164	149	G
Acetophenone, 4-hydroxy-3-methoxy- (Acetovanillone)	28,569	1387	151	166	G
***Phenol, 2,6-dimethoxy- (Syringol)**	**28,611**	**1389**	**154**	**139**	**S**
Phenol, 3,4-dimethoxy-	28,884	1390	154	139	S
Phenol, 2-methoxy-4-propyl- (Propylguaiacol)	28,894	1392	137	166	G
Benzaldehyde, 4-hydroxy-	29,287	1398	121	122	H
Phenol, 4-propyl-	29,415	1399	107	77	H
4-Ethylcatechol	29,707	1410	123	138	PolyOH
Phenol, 2-methoxy-5-(1-propenyl)-	30,173	1425	164	149	G
Phenol, 2-methoxy-3-(1-propenyl)- (Isoeugenol)	30,487	1434	164	149	G
Phenol, 2-methoxy-4-(1-propenyl)- (Isoeugenol)	30,777	1440	164	149	G
Benzaldehyde, 4-hydroxy-3-methoxy- (Vanillin)	30,868	1440	151	152	G
Phenol, 5-methoxy-2,3-dimethyl-	31,574	1442	152	137	G
Phenol, 2-methoxy-4-propenyl- (cis-Isoeugenol)	32,711	1487	164	149	G
***Phenol, 4-methyl, 2,6-dimethoxy- (4-methylsyringol)**	**32,974**	**1488**	**168**	**153**	**S**
Benzaldehyde, 2-hydroxy-6-methyl-	33,007	1490	136	90	G
Phenol, 2-methoxy-4-propyl-	33,468	1497	137	166	G
Phenol, 2-methoxy-4-acetyl	34,583	1526	151	166	G
Acetylguaiacol isomer	34,809	1532	166	151	G
Benzoic acid, 4-hydroxy-3-methoxy-, methyl ester (Methyl vanillate)	35,809	1532	151	182	G

4-ethyl-2,6-dimethoxy-phenol (4-ethyl-syringol)	36,174	1572	167	182	S
4-(2-Hydroxyethyl)-2-methoxyphenol	36,431	1577	137	180	G
Ethanone, 1-(4-hydroxy-3-methoxyphenyl)-	37,184	1594	151	166	G
Phenol, 4-ethenyl, 2,6-dimethoxy (4-vinylsyringol)	37,825	1615	180	165	S
4-((1E)-3-Hydroxy-1-propenyl)-2-methoxyphenol	38,152	1624	137	124	G
Benzeneacetic acid, 4-hydroxy-3-methoxy-, methyl ester	38,421	1632	137	196	G
4-Hydroxy-2-methoxycinnamaldehyde	38,486	1634	178	135	G
propano 3-methoxy-4-hydroxyphenone	38,518	1634	151	123	G
Ethanone, 1-(4-hydroxy-3-methoxyphenyl)- (Apocynin)	38,815	1638	151	123	G
Phenol, 4-(2-propenyl), 2,6-dimethoxy- (4-allylsyringol)	39,316	1656	194	91	S
Phenol, 4-propyl, 2,6-dimethoxy- (4-propylsyringol)	39,566	1663	167	196	S
4-n-Propylresorcinol	39,828	1665	123	152	PolyOH
Phenol, 4-(3-hydroxy-1-propenyl)-	40,793	1683	107	150	G
(4-Hydroxy-3-methoxyphenyl)acetic acid (Homovanillic acid)	41,398	1702	137	182	G
Phenol, 4-(2-propenyl), 2,6-dimethoxy-, cis-	41,523	1708	194	91	S
Benzaldehyde, 4-hydroxy-3,5-dimethoxy- (Syringaldehyde)	41,837	1717	182	93	S
4-((1E)-3-Hydroxy-1-propenyl)-2-methoxyphenol	42,108	1728	137	180	G
1-Propanone, 3-hydroxy-1-(4-hydroxy-3-methoxyphenyl)-	42,727	1744	151	108	G
3-Methoxy-4-hydroxybenzalacetone	42,816	1748	149	192	G
Phenol, 2,6-dimethoxy-4-(2-propenyl)- (4-propenylsyringol- trans)	43,237	1762	194	91	S
1-(4-Hydroxy-3,5-dimethoxyphenyl)-ethanal (Homosyringaldehyde)	43,467	1765	167	196	S
Acetic acid, (4-hydroxy-3-methoxyphenyl)-, methyl ester (Methyl homovanillate)	44,258	1785	137	196	G
2-Propenal, 3-(4-hydroxy-3-methoxyphenyl)- (Coniferyl aldehyde)	44,502	1793	178	147	G
Acetophenone, 4-hydroxy-3,5-dimethoxy- (Acetosyringone)	44,533	1796	181	196	S
2-Propen-1-ol, 3-(4-hydroxy-3-methoxyphenyl)- (Coniferyl alcohol)	44,853	1802	137	180	G
2-Butanone, 4-(4-hydroxy-3-methoxyphenyl)- (Vanillylacetone; Gingerone)	45,502	1825	137	194	G
Benzoic acid, 4-hydroxy-3,5-dimethoxy-, methyl ester (Syringic acid, methyl ester)	45,793	1836	181	212	S
1-(4-Hydroxy-3,5-dimethoxyphenyl)-2-propanone (Syringyl acetone)	45,988	1837	167	210	S
3-(4-Hydroxy-3,5-dimethoxyphenyl)-1-propanal	47,631	1885	167	182	S
1-Propanone, 1-(4-hydroxy-3,5-dimethoxyphenyl) (Propiosyrongone)	47,757	1892	181	153	S
1-Propen-2-al, 3-(4-hydroxy-3,5-dimethoxyphenyl) (Propenylsyringone)	48,063	1896	208	177	S
1-(4-Hydroxy-3,5-dimethoxyphenyl)-1-propanol (Dihydrosinapyl alcohol)	50,283	1959	168	212	S
2-Propen-1-ol, 3-(4-hydroxy-3,5-dimethoxyphenyl) (Sinapyl alcohol, cis)	50,961	1982	167	210	S
Sinapic aldehyde	53,266	2052	208	177	S
2-Propen-1-ol, 3-(4-hydroxy-3,5-dimethoxyphenyl) (Sinapyl alcohol, trans)	53,499	2064	210	182	S

* main products in each group

Table S2. Effect of temperature on the thermal decomposition

Units, %	Temperature, °C						HSQC NMR
	400	350	300	250	**200**	150	
H	0.4 ± 0.1	0.2 ± 0.1	0.3 ± 0.1	0.3 ± 0.1	**0.8 ± 0.3**	1.1 ± 0.4	**1.3 ± 0.1**
G	25.0 ± 3.0	25.6 ± 3.0	25.4 ± 3.0	26.6 ± 3.2	**30.1 ± 3.6**	31.0 ± 3.7	**29.5 ± 0.9**
S	74.6 ± 3.8	74.2 ± 3.8	74.3 ± 3.8	73.1 ± 3.8	**68.5 ± 3.5**	67.9 ± 3.5	**69.8 ± 1.8**
Poly-OH	0.7	0.5	0.4	0.3	**0.3**	0.0	-

Table S3. Effect of thermal desorption duration

Units, %	Process duration, min					HSQC NMR
	5	10	**20**	**30**	60	
H	0.5 ± 0.2	0.6 ± 0.2	**0.4 ± 0.1**	**0.4 ± 0.1**	0.4 ± 0.1	**1.3 ± 0.1**
G	31.9 ± 3.8	32.7 ± 3.9	**31.1 ± 3.7**	**28.6 ± 3.4**	29.1 ± 3.4	**29.5 ± 0.9**
S	67.6 ± 3.5	66.7 ± 3.4	**68.6 ± 3.5**	**71.0 ± 3.6**	70.5 ± 3.6	**69.8 ± 1.8**
Poly-OH	0.11	0.08	**0.26**	**0.33**	0.25	-

Table S4. Effect of heating rate in the DS-mode

Units, %	Heating rate, °C/min								HSQC NMR
	20	40	60	80	**100**	**120**	250	800	
H	0.8±0.1	0.8±0.1	0.9±0.1	0.7±0.1	**0.8±0.1**	**0.9±0.1**	0.9±0.1	1.0±0.2	**1.3 ± 0.1**
G	26.1±1.2	26.5±1.3	28.6±1.4	28.4±1.4	**28.7±1.4**	**30.1±1.4**	30.2±1.4	31.6±1.5	**29.5 ± 0.9**
S	73.1±1.2	72.7±1.2	70.5±1.2	70.9±1.2	**70.5±1.2**	**69.3±1.2**	68.8±1.2	67.3±1.1	**69.8 ± 1.8**

Table S5. Effect of sample weight in the DS-mode

Units, %	Sample weight, mg			HSQC NMR
	0.2	0.4	0.9	
H	0.8 ± 0.1	0.8 ± 0.1	0.8 ± 0.1	1.3 ± 0.1
G	29.1 ± 1.4	28.7 ± 1.4	27.0 ± 1.3	29.5 ± 0.9
S	70.0 ± 1.2	70.5 ± 1.2	72.2 ± 1.2	69.8 ± 1.8

Table S6. Peak shape parameters

Parameter *	Sample weight, mg		
	0.2	0.4	0.9
Phenol, 4-methyl, 2,6-dimethoxy- (4-methylsyringol)			
A/H	6.849	8.549	13.316
FWHM	0.101	0.133	0.218
AsF	0.614	0.395	0.226
TF	0.862	0.709	0.620
Phenol, 2,6-dimethoxy- (Syringol)			
A/H	8.549	8.224	12.360
FWHM	0.107	0.126	0.204
AsF	1.706	0.574	0.264
TF	1.629	0.885	0.639

* A/H - ratio of the peak area to the peak height; FWHM - full width at half maximum; AsF - peak asymmetry factor; TF - peak tailing factor

Table S7. Effect of temperature in the DS-mode

Units, %	Temperature, °C							HSQC NMR
	700	550	**450**	**400**	350	300	250	
H	4.9±0.7	4.8±0.7	**2.0±0.3**	**0.8±0.1**	0.5±0.1	0.3±0.1	0.2±0.1	**1.3 ± 0.1**
G	30.7±1.5	30.6±1.5	**29.3±1.4**	**28.7±1.4**	27.2±1.3	27.2±1.3	23.3±1.1	**29.5 ± 0.9**
S	64.3±1.1	64.6±1.1	**68.8±1.2**	**70.5±1.2**	72.3±1.2	72.5±1.2	76.5±1.3	**69.8 ± 1.8**
Poly-OH	8.1	7.8	**5.4**	**2.3**	0.6	0.0	0.0	-

Table S8. Effect of temperature in the SS-mode.

Units, %	Temperature, °C							HSQC NMR
	700	550	450	**400**	**350**	300	250	
H	74.7±19.4	4.2±1.1	2.2±0.6	**1.0±0.3**	**0.4±0.1**	0.2±0.1	0.3±0.1	**1.3 ± 0.1**
G	21.6±1.8	34.0±2.9	32.8±2.8	**31.6±2.7**	**29.4±2.5**	30.7±2.6	27.1±2.3	**29.5 ± 0.9**
S	3.7±0.1	61.8±2.2	65.0±2.3	**67.3±2.4**	**70.2±2.5**	69.0±2.5	72.6±2.6	**69.8 ± 1.8**
Poly-OH	48.3	6.1	4.4	**4.6**	**1.2**	0.0	0.0	-
